# The roles of shared vs. distinctive conceptual features in lexical access

**DOI:** 10.3389/fpsyg.2014.01014

**Published:** 2014-09-16

**Authors:** Harrison E. Vieth, Katie L. McMahon, Greig I. de Zubicaray

**Affiliations:** ^1^School of Psychology, University of QueenslandBrisbane, QLD, Australia; ^2^Centre for Advanced Imaging, University of QueenslandBrisbane, QLD, Australia

**Keywords:** lexical access, competition, semantic interference, picture naming, shared features, distinctive features

## Abstract

Contemporary models of spoken word production assume conceptual feature sharing determines the speed with which objects are named in categorically-related contexts. However, statistical models of concept representation have also identified a role for *feature distinctiveness*, i.e., features that identify a single concept and serve to distinguish it quickly from other similar concepts. In three experiments we investigated whether distinctive features might explain reports of counter-intuitive semantic facilitation effects in the picture word interference (PWI) paradigm. In Experiment 1, categorically-related distractors matched in terms of semantic similarity ratings (e.g., *zebra* and *pony*) and manipulated with respect to feature distinctiveness (e.g., a *zebra* has stripes unlike other equine species) elicited interference effects of comparable magnitude. Experiments 2 and 3 investigated the role of feature distinctiveness with respect to reports of facilitated naming with part-whole distractor-target relations (e.g., a *hump* is a distinguishing part of a CAMEL, whereas *knee* is not, vs. an unrelated part such as *plug*). Related part distractors did not influence target picture naming latencies significantly when the part denoted by the related distractor was not visible in the target picture (whether distinctive or not; Experiment 2). When the part denoted by the related distractor was visible in the target picture, non-distinctive part distractors slowed target naming significantly at SOA of −150 ms (Experiment 3). Thus, our results show that semantic interference does occur for part-whole distractor-target relations in PWI, but only when distractors denote features shared with the target and other category exemplars. We discuss the implications of these results for some recently developed, novel accounts of lexical access in spoken word production.

## Introduction

A large empirical literature on object naming has demonstrated that speakers are influenced by the activation of concepts related to the object they intend to name. For example, when objects are presented in categorically related vs. unrelated contexts, naming latencies are typically slower (e.g., Rosinski, 1977; Lupker, 1979; Kroll and Stewart, 1994). Virtually all accounts of spoken word production assume that these semantic context effects occur due to the co-activation of conceptual features shared among categorically related objects. However, there is considerable disagreement among accounts as to the consequences of this *conceptual feature overlap* for the production system (e.g., Dell and O'Seaghdha, 1992; Caramazza, 1997; Levelt et al., 1999; Goldrick and Rapp, 2002; Mahon et al., 2007; Rahman and Melinger, 2009).

Semantic context effects are induced successfully in a number of experimental naming paradigms. For example, in the picture-word interference (PWI) paradigm, in which participants ignore a distractor word while naming a target picture, slower naming latencies are observed reliably when distractors (e.g., *wolf)* are category coordinates of the target picture (e.g., DOG) compared to unrelated distractors (e.g., *book*; Schriefers et al., 1990; Levelt et al., 1991; La Heij and van den Hof, 1995). This effect is known as *semantic interference* and has been interpreted as evidence supporting a competitive lexical selection mechanism in some spoken word production models (Starreveld and La Heij, 1996; Levelt et al., 1999; Rahman and Melinger, 2009). However, non-competitive lexical selection mechanisms have also been proposed to explain the effect (Caramazza, 1997; Mahon et al., 2007).

The *lexical selection by competition* account assumes that naming latencies are a function of the number of active lexical candidates and their activation levels. For instance, if the target concept “HORSE” is activated, related animal category concepts such as *pony, cow* etc. also become activated due to conceptual feature overlap, and this activation spreads to their lexical representations (e.g., Collins and Loftus, 1975). This account explains the semantic interference effect in the PWI paradigm in terms of the categorically related distractor increasing the activation level of an existing lexical competitor, slowing target selection compared to an unrelated distractor word that activates a concept that was not activated by the target picture.

Some PWI studies have demonstrated that conceptual feature overlap might not necessarily induce semantic interference. Costa et al. (2005) reported that naming latencies were *facilitated* using “part-whole” distractor-target pairs (*bumper*-CAR), a result confirmed by Muehlhaus et al. (2013). Further, in two PWI experiments using two different methods of determining semantic overlap, Mahon et al. (2007; Experiments 5 and 7) showed target naming latencies (e.g., HORSE) were facilitated for semantically “close” distractors (e.g., *zebra*) compared to semantically “far” distractors (e.g., *whale*). Mahon et al. (2007) argued that part and semantically close distractors should have higher conceptual-lexical activation levels due to sharing features with the target and thus be stronger competitors according to the competitive lexical selection account. They therefore proposed a post-lexical, non-competitive, *response exclusion* account of lexical selection. According to this account, conceptual feature overlap between distractor and target invariably induces semantic priming. Semantic interference in PWI instead reflects the extent to which the distractor is a relevant response to the task of naming the target picture. If the distractor is a relevant response to the target (e.g., another animal), a post-lexical decision mechanism must take more time to clear it from an articulatory buffer. Further, the account predicts the part-whole facilitation effect in PWI (Costa et al., 2005), as the “part” (e.g., *bumper*) is not a relevant response to the target picture (e.g., CAR).

Rahman and Melinger (2009) modified the competitive lexical selection account to explain part-whole and semantic distance facilitation effects in the PWI paradigm. According to their *swinging lexical network* model, feature-overlap between targets and distractors invariably produces semantic priming *and* interference. A net result of interference or facilitation depends upon the pattern of activation within the network. If shared features between the target and distractor activate a cohort of within-category lexical competitors, this creates one-to-many competition, and the net result is interference. Facilitation results when feature overlap does not spread to many lexical competitors, causing one-to-one competition. As distractors that are parts of whole objects do not spread activation to other related concepts, they produce one-to-one rather than one-to-many competition, and the net result is facilitation. Similarly, facilitation for semantically close distractor-target pairings is attributed to stronger priming due to feature overlap coupled with activation of a narrower category cohort of competitors (e.g., HORSE and *zebra* will activate only members of the equine category), contrasted with weaker priming and activation of a larger cohort for semantically far distractors (e.g., HORSE and *whale* will activate the broader category of animals).

However, more recent research has failed to elicit facilitation effects with similar stimuli. For example, Piai et al. (2011) noted that part-whole facilitation might instead be driven by strong associative links between the part distractor and its corresponding whole. Previous research has shown naming latencies are facilitated when targets are paired with distractors that are associates (e.g., SHIP-port; La Heij et al., 1990; Alario et al., 2000). Muehlhaus et al. (2013) selected part-whole stimuli that were strong associates using cue-target free association norms. Consistent with this explanation, Sailor and Brooks (2014) found that part-distractors produced facilitation only when they were associated with the target. When not associated with the target, part-distractors produced an interference effect compared to parts unrelated to the target object (Experiments 1 and 3). Further, Sailor and Brooks (2014) were unable to replicate the findings from Costa et al.'s (2005) second experiment using identical materials. In two separate PWI experiments, Vieth et al. (2014) were likewise unable to replicate the facilitation effect reported by Mahon et al. (2007; Experiment 7) using near identical stimuli based on feature production norms (McRae et al., 2005). Instead, they found reliably greater interference for distractors that shared more features with the target.

Might there be another explanation for the (albeit equivocal) reports of feature overlap producing facilitation effects in PWI? To date, all accounts have emphasized feature-overlap between concepts. However, there is considerable behavioral evidence, supported by computational simulations, that distinctive features are activated differentially—and perhaps preferentially—to shared features (Randall et al., 2004; Cree et al., 2006; Grondin et al., 2009). Distinctive features can be defined as features that are (ideally) a perfect cue to a concept, distinguishing it from other related concepts, or in terms of narrowing a contrast set. For instance, the feature “has a *talon*,” is likely to narrow a contrast set to <birds of prey> (see Cree et al., 2006). As Grondin et al. (2009) note, distinctive features “make it easier to respond when the task requires distinguishing an item from among similar items, such as when naming the picture of an object” (p. 6, see also Cree et al., 2006; Taylor et al., 2012).

An examination of the stimuli employed by Mahon et al. (2007) in their Experiment 5 indicates that 17/20 of the *close* target-distractor pairings involved *at least one* distinguishing feature (e.g., HORSE-zebra). These stimuli were selected based on semantic similarity ratings from an independent sample of participants. Past research has shown that similarity ratings tend to emphasize the importance of shared features while de-emphasizing distinguishing features (e.g., Medin et al., 1995; Kaplan and Medin, 1997). For example, the *coincidence effect* refers to the finding that two items (e.g., *horse* and *zebra*) that are semantically close due to feature overlap (e.g., *equine animal, has legs, has a tail, etc*.) yet differ due to a distinguishing feature (e.g., *has stripes*) will tend to receive a higher similarity rating than do two items that share a similar number of semantic features yet only differ modestly (e.g., *horse* and *donkey*). Thus, if distinctive features have a privileged role during conceptual processing (Cree et al., 2006), in that they are activated more quickly and/or strongly than shared features, this might explain why Mahon et al. (2007) (Experiment 5) observed facilitation for their semantically close distractors that contained a high proportion of distinctive features, despite also sharing a number of features with the target pictures.

A similar examination of the part-whole stimuli employed by Costa et al. (2005) indicates that many are distinctive parts of their targets according to published feature production norms (McRae et al., 2005; e.g. PERISCOPE-*submarine*; SINK-*drain*). Other pairings likewise appear distinguishing (e.g., CHURCH-*pew*; AMBULANCE-*stretcher*). As Costa et al. (2005; also Mahon et al., 2007) note, the activation-level of a part distractor should be raised when presented in conjunction with a target picture of the whole object to which it refers, due to feature overlap, thus making it a more potent competitor according to the lexical selection by competition account. However, a part that is a distinctive feature and so potentially a perfect cue to the target concept should elicit less lexical-level activation than a part that is shared with other objects, due to less activation spreading at the conceptual level. This might explain why some studies observed facilitation with part-whole distractor-target pairings while others observed interference (e.g., Sailor and Brooks, 2014).

**Figure 1:**
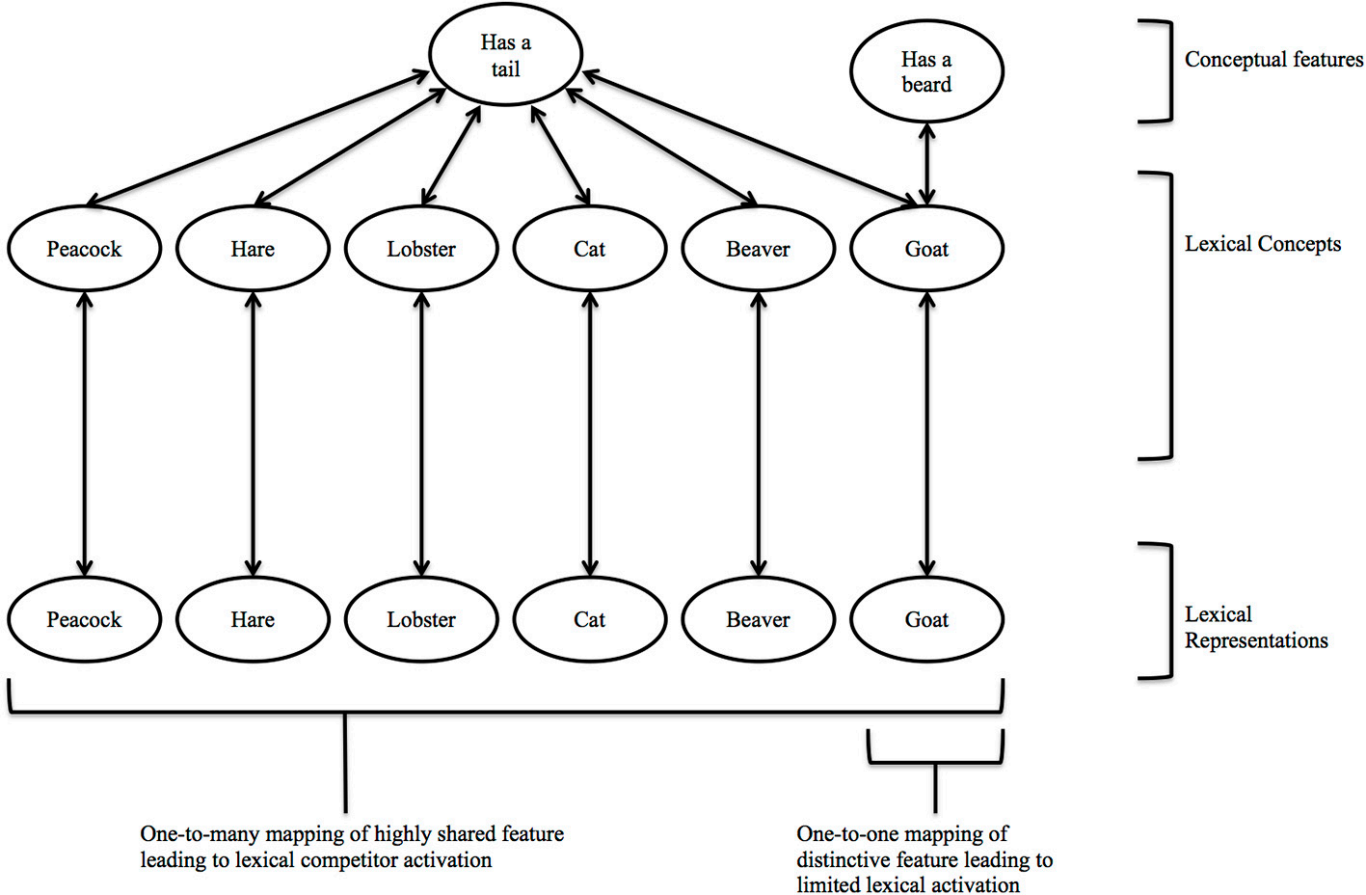
**A depiction of how a distinctive feature (*beard* for GOAT) might operate within the lexical-conceptual network compared with a shared feature (*tail*)**. Activation of *beard* spreads activation only to the lexical concept it is linked to, facilitating its production, whereas activation of a shared feature like *tail* spreads activation to a larger lexical cohort (e.g., 39 animals have a tail according to the (McRae et al., 2005) feature norms), inducing competition with the target utterance.

Thus, feature distinctiveness might be an important factor influencing the polarity of semantic effects in PWI paradigms. If so, accounts of semantic facilitation effects in spoken word production models would need to be modified to account for preferential processing of distinctive features. Conceivably, both post-lexical and swinging lexical network accounts of PWI effect could be modified to accommodate potential facilitatory effects of distinctive features in terms of stronger semantic priming, the former by assuming that the processing of distinctive distractors is privileged such that they enter the articulatory buffer earlier and are excluded accordingly, while the latter model could assume that distinctive features result in one-to-one rather than one-to-many competition at the lexical level due to their activating only the relevant target concept (see Figure 1), and so the net effect is semantic priming.

In this study, we report three PWI experiments examining effects of shared and distinctive distractor features. Experiment 1 manipulated distinctive distractor features while controlling for shared features, with the aim of determining whether the former might be responsible for eliciting a facilitation effect with categorical distractor-target relations (e.g., Mahon et al., 2007; Experiment 5). Experiments 2 and 3 investigated the role of feature distinctiveness with respect to part-whole distractor-target relations (e.g., a *hump* is a distinguishing part of a CAMEL, whereas *knee* is not, vs. an unrelated part such as *plug*). In all three experiments, targets and distractors were constructed so as to have minimal associative relations (e.g., Sailor and Brooks, 2014).

## Experiment 1

Experiment 1 tested whether *feature distinctiveness* might facilitate naming of categorically-related distractor-target pairings, as they are known to speed simple picture naming (e.g., Grondin et al., 2009). Past research has shown that similarity ratings tend to weight shared features as more important, with two items (e.g., *horse* and *zebra*) matching on one dimension (e.g., *equine animal*) yet differing considerably on another (e.g., *stripes*) tending to receive a higher similarity rating than two items that differ modestly (e.g., *horse and donkey*; Medin et al., 1995; Kaplan and Medin, 1997). As we noted in the Introduction to this paper, an examination of the *close* distractor-target pairings in Mahon et al.'s (2007) Experiment 5 revealed the majority involved distinguishing features (e.g., HORSE-*zebra*) according to feature production norms. Thus, distinguishing features might be responsible for the polarity reversal they observed. Experiment 1 therefore employed a set of target-distractor materials that manipulated distinctive features while controlling for semantic similarity.

### Participants

Participants were 50 students enrolled in first-year psychology courses at the University of Queensland. All were native English speakers. Each participant gave informed consent in accordance with the protocol approved by the Behavioral and Social Sciences Ethical Review Committee of the University of Queensland and was compensated with course credit.

### Design

Experiment 1 was a 2 × 2 × 2 mixed design. Independent variables within-participants were semantic relation (*semantically related, unrelated)*, and distinctiveness (distinctive, non-distinctive) and SOA between-participants (−160 and 0 ms). These SOAs were selected based on the findings of significant facilitation effects in Mahon et al.'s (2007) Experiments 5 (0 ms) and 7 (−160 ms). Twenty-five participants were randomly assigned to each SOA.

### Materials

Twenty target pictures and 40 distractor words were selected via a ratings study. Pictures were black-and-white line drawings, the majority of which were selected from normative picture databases (Cycowicz et al., 1997; Bonin et al., 2003; Szekely et al., 2004) with remaining items from the internet. The distractors were split into two sets of 20 categorically related items that were matched in terms of semantic similarity to the targets. In one of these sets (*similar-plus-distinctive*), each distractor additionally had at least one feature dimension rated as distinguishing it from the target, despite being matched in overall rated similarity. By way of example, a *semantically similar* pairing was PIGEON-*sparrow* while the corresponding *similar-plus-distinctive* pairing was PIGEON-*canary*. In order to reduce the number of related trials in the experiment to approximately 50%, two unrelated distractor conditions were created by re-pairing each distractor with an unrelated target picture (following Mahon et al., 2007; see Appendix A).

In order to create the *semantically similar* and *similar-plus-distinctive* target-distractor pairings, we performed two separate ratings studies. In the first, a group of 37 participants, none of whom participated in the PWI experiment, performed semantic similarity and dissimilarity judgments on a list comprising 72 word triplets, each triplet consisting of a target and two categorically related distractors. Targets were paired with each distractor separately on different trials. Participants were required to rate target-distractor word pairs presented in random order for semantic similarity (“how related are the two concepts denoted by the words”) on a scale of 1 to 7 (1 = unrelated, 7 = highly related) following Mahon et al. (2007). Subsequently, the participants were presented with the word triplets, again in random order, and instructed to select the distractor concept that differed most from the target and nominate the distinguishing feature. In the second ratings study, another group of 11 participants, none of whom participated in the first rating study or the PWI experiment, rated each word for imageability (“the ability to form a picture of the word's referent in your mind”) following Mahon et al. (2007). Ratings were made on a scale of 1 to 7 (1 = not imageable, 7 = highly imageable).

The sets of 20 *semantically similar* and 20 *similar-plus-distinctive* distractors were thus created using triplets in which both distractors had been rated as highly similar to the target. The *similar-plus-distinctive* distractors were selected according to the consistency with which a distinguishing feature dimension had been nominated across participants (criterion > 70%). Distractors in both sets were also matched according to imageability ratings, frequency, number of morphemes, syllables, and phonemes, word length, orthographic (OLD) and phonological Levenshtein Distance (PLD) (see Table 1; Balota et al., 2007). A series of *t*-tests demonstrated no significant differences between semantically related conditions on similarity to target *t*_(38)_ = 1.006, *p* = 0.32, imageability *t*_(38)_ = 1.68, *p* = 0.10, word length *t*_(38)_ = 0.21, *p* = 0.84, frequency *t*_(38)_ = 0.17, *p* = 0.87, OLD *t*_(38)_ = 0.17, *p* = 0.87, PLD *t*_(38)_ = 0.71, *p* = 0.71, number of phonemes *t*_(38)_ = 0.61, *p* = 0.54, number of syllables *t*_(38)_ = 0, *p* = 1, and number of morphemes *t*_(38)_ = 1.24, *p* = 0.22. Trials were randomized using Mix software (van Casteren and Davis, 2006) with the constraints that two presentations of the same picture were always interceded by at least five different pictures, and no more than two consecutive trials were of the same distractor type. One unique list per participant was generated.

**Table 1:** **Matching variables for the stimuli in Experiment 1**.

	**Distractor Type**	
	**Similar**	**Similar-plus-distinctive**
Rated similarity to target	5.33 (0.44)	5.20 (0.39)
Imageability	6.16 (0.65)	6.44 (0.35)
OLD	2.08 (0.74)	2.12 (0.76)
PLD	1.97 (0.82)	2.07 (0.86)
No. Phonemes	4.7 (1.34)	4.95 (1.23)
No. Syllables	1.75 (0.55)	1.75 (0.64)
No. Morphemes	1.1 (0.31)	1.25 (0.44)

*Standard Deviations are in parentheses*.

*OLD, Orthographic Levenshtein Distance; PLD, Phonological Levenshtein Distance*.

### Apparatus

Stimuli presentation, response recording and latency measurement (i.e., voice key) were accomplished via the Cogent 2000 toolbox extension (www.vislab.ucl.ac.uk/cogent_2000.php) for MATLAB (2010a, MathWorks, Inc.) using a personal computer equipped with a noise-canceling microphone (Logitech, Inc.). The same apparatus was used in all subsequent experiments.

### Procedure

Participants underwent pre-experimental familiarization with the target pictures by naming each three times in random order. The first presentation was accompanied by the target's proper name printed below, with subsequent presentations only displaying the picture. Each experimental trial commenced with the participant pressing the space bar following the presentation of a question mark (?) at center-screen. Trials began by presenting a fixation cross center-screen for 500 ms, followed by a 50 ms blank screen. The distractor word appeared at −160 or 0 ms SOA relative to target presentation. Distractor words appeared randomly either above or below targets and counterbalanced across trials/conditions. Stimuli remained onscreen for 3000 ms or until the participant made a verbal response. A question mark presented centrally then indicated that the participant could proceed to the next trial via space bar press.

### Results and discussion

Trials on which the voice key failed to detect a response (0.01%) were discarded as were latencies below 250 ms or above 2000 ms (2.5%). Latencies deviating more than 2.5 standard deviations from within-participant, within-condition means were excluded from analysis (5.7%). Errors were classified according to whether the participant hesitated during naming (i.e., dysfluencies) or misidentified the target, and due to their low frequency (1.6%) were not subjected to analysis. Mean naming latencies and error rates are summarized in Table 2.

**Table 2:** **Experiment 1: Naming Latencies (in Milliseconds), 95% Confidence Intervals (CI), and Error rates (E%) by Type of Distractor and SOA**.

	**Distractor condition**			
	**Semantically similar**	**Similar-plus-distinctive**	**Unrelated (similar)**	**Unrelated (similar-plus-distinctive)**
**SOA − 160 ms**				
Mean	784	777	760	756
*CI*	±47	±44	±46	±45
*E%*	1	2	1.2	2
**SOA 0 ms**				
Mean	801	813	794	791
*CI*	±47	±44	±46	45
*E%*	1.8	1.2	1	2.2

Data were subjected to repeated measures ANOVAs with participants and items as random factors (*F*_1_ and *F*_2_, respectively). There was a significant main effect of distractor relation, *F*_1(1, 48)_ = 8.40, *p* = 0.006, partial η^2^ = 0.15, *F*_2(1, 38)_ = 14.41, *p* = 0.001, partial η^2^ = 0.28, yet no significant effect of distinctiveness *F*_1(1, 48)_ < 1, *p* = 0.963, partial η^2^ = 0.00, *F*_2(1, 38)_ < 1, *p* = 0.978, partial η^2^ = 0.00. The effect of SOA was not significant by participants *F*_1(1, 48)_ < 1, *p* = 0.326, partial η^2^ = 0.02, although was significant by items *F*_2(1, 38)_ = 6.21, *p* = 0.017, partial η^2^ = 0.14, with naming latencies faster at SOA −160 ms. There were no significant interactions between distractor relation and either distinctiveness, *F*_1(1, 48)_ < 1, *p* = 0.546, partial η^2^ = 0.01, *F*_2(1, 38)_ < 1, *p* = 0.469, partial η^2^ = 0.01, or SOA, *F*_1(1, 48)_ < 1, *p* = 0.561, partial η^2^ = 0.01, *F*_2(1, 38)_ < 1, *p* = 0.601, partial η^2^ = 0.01.

Separate analyses were conducted within each SOA. At −160 ms SOA, there was a significant effect of distractor relation, *F*_1(1, 24)_ = 7.47, *p* = 0.012, partial η^2^ = 0.24, *F*_2(1, 19)_ = 9.46, *p* = 0.006 partial η^2^ = 0.33. However, there was no significant effect of distractor distinctiveness *F*_1(1, 24)_ < 1, *p* = 0.537, partial η^2^ = 0.02, *F*_2(1, 19)_ < 1, *p* = 0.409, partial η^2^ = 0.04, or interaction between distinctiveness and relation, *F*_1(1, 24)_ < 1, *p* = 0.760, partial η^2^ = 0.00, *F*_2(1, 19)_ < 1, *p* = 0.792, partial η^2^ = 0.00. At 0 ms SOA, there was no significant effect of distractor relation by participants *F*_1(1, 24)_ = 2.25, *p* = 0.147, partial η^2^ = 0.09, although the effect was significant by items *F*_2(1, 19)_ = 5.28, *p* = 0.033 partial η^2^ = 0.22. Again, there was no significant effect of distinctiveness, *F*_1(1, 24)_ < 1, *p* = 0.473, partial η^2^ = 0.02, *F*_2(1, 19)_ = 1.47, *p* = 0.240, partial η^2^ = 0.07 and no interaction, *F*_1(1, 24)_ = 1.52, *p* = 230, partial η^2^ = 0.06, *F*_2(1, 19)_ = 1.21, *p* = 0.285, partial η^2^ = 0.06.

Follow up comparisons investigated the significant main effects of distractor relation found at each SOA. At −160 ms SOA, related distractor-target pairs were named significantly slower than unrelated pairs, *t*_1(24)_ = 2.73, *p* = 0.012, *M*_diff_ = 23 ms, 95% *CI* = ±17, *t*_2(19)_ = 3.08, *p* = 0.006, *M*_diff_ = 21 ms, 95% *CI* = ±14. At 0 ms SOA, related distractor-target pairs were named significantly slower than unrelated pairs, *t*_2(19)_ = 2.30, *p* = 0.033, *M*_diff_ = 16 ms, 95% *CI* = ±14.

Contrary to our prediction, categorically related distractors with distinguishing features did not influence picture naming latencies differentially: both *similar* and *similar-plus-distinctive* distractors elicited comparable interference compared to the matched unrelated distractors at each SOA. This result indicates that distinguishing features are unlikely to be responsible for semantic facilitation effects observed for categorically related distractors and targets in some PWI experiments using high proportions of distractor-target pairings with distinguishing features (e.g., Mahon et al., 2007; Experiment 5). Moreover, they indicate that conceptual feature overlap is the predominant factor influencing naming latencies in the PWI paradigm when distractors and targets are categorically related. However, the results of Experiment 1 are not informative with respect to the role of distinctive features when distractors are *not* category coordinates of the target picture, as is the case with part-whole relations (e.g., Costa et al., 2005). This latter scenario is explored in Experiment 2.

## Experiment 2

As noted in the Introduction, Costa et al. (2005) stimuli included distractors that denoted distinctive parts of their targets (e.g., *periscope*-SUBMARINE) according to feature production norms (McRae et al., 2005). In the absence of a categorical relation, part-whole distractor-target pairings represent a context in which a distinctive feature has a one-to-one relationship with a target picture concept that might facilitate its identification via semantic priming (e.g., Taylor et al., 2012), whereas the relationship of a non-distinctive feature is less clear as it is shared among other objects. Experiment 2 therefore employed a set of materials that manipulated distinctive vs. non-distinctive parts of target objects, while ensuring associative relations were minimal (e.g., Piai et al., 2011; Sailor and Brooks, 2014).

### Participants

Twenty-nine students from the University of Queensland participated in this study. All were native English speakers. Each participant gave informed consent and was compensated with course credit.

### Research design

Experiment 2 was a 2 × 2 × 3 repeated measures design, with target picture naming latencies being the dependent variable. The three independent variables were distractor part-relation (related, unrelated), distinctiveness (distinctive, non-distinctive), and SOA (−150, 0, or +150 ms), using a within-participants design, following Sailor and Brooks' (2014) findings at SOAs of −150 and 0 ms.

### Materials

**Figure 2:**
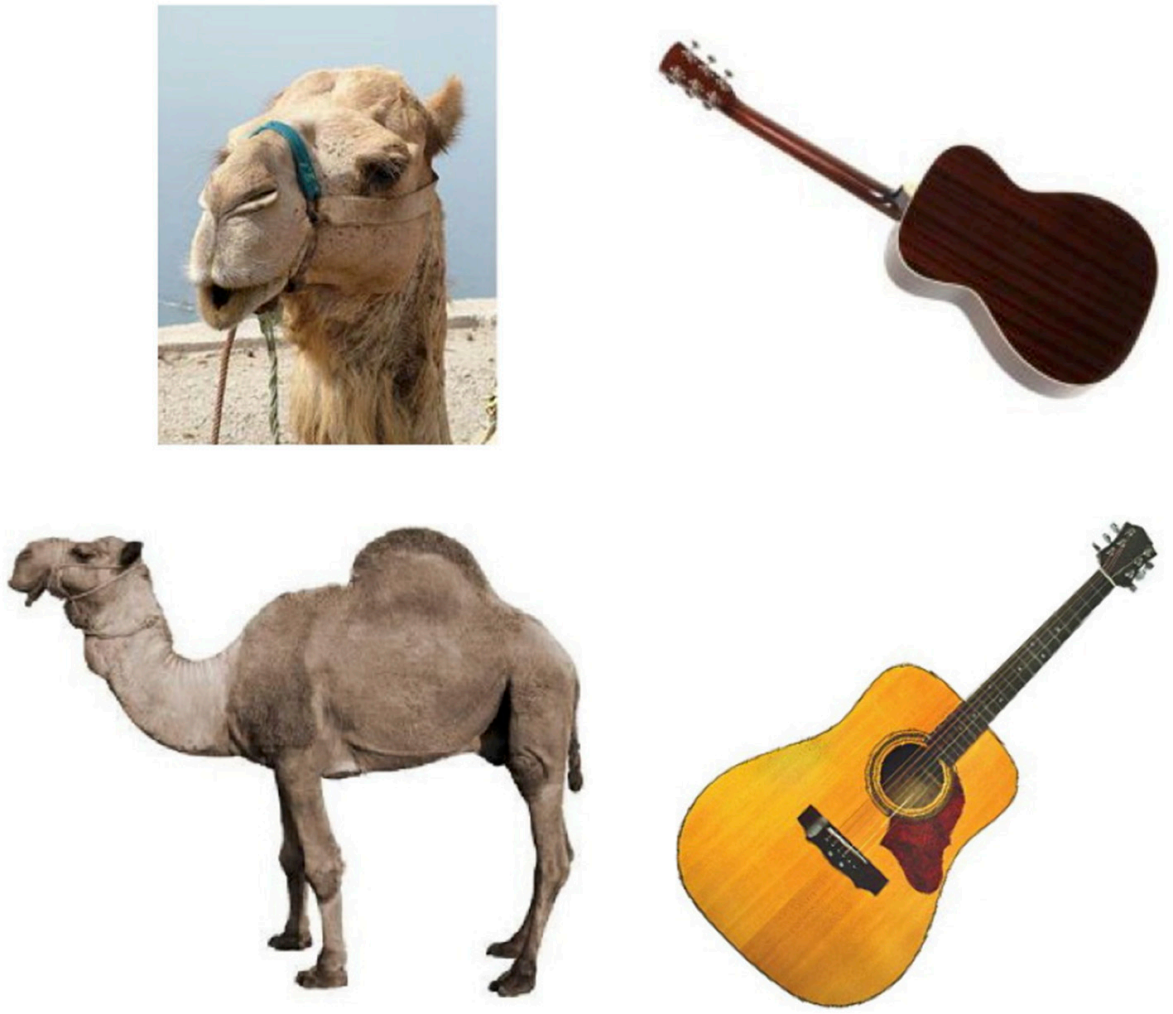
**Examples of target picture stimuli CAMEL (left) and GUITAR (right) for Experiment 2 (top row) and Experiment 3 (bottom row)**. In the target pictures for Experiment 2, distractor parts (*hump* and *knee* for CAMEL, *hole* and *fret* for GUITAR) are not visible following Costa et al. (2005).

Twenty-four target pictures and 48 distractors were selected according to published feature production norms (McRae et al., 2005; see Appendix B). Pictures were color photographs sourced from normative databases (e.g., Adlington et al., 2009; Moreno-Martínez and Montoro, 2012) and the internet. Distinctive features were determined via the “distinctiveness” measure in the McRae et al. (2005) norms, defined as the inverse of the number of concepts in which that feature occurs in the norms. Therefore, those features with high scores occur less often between different concepts and are thus more distinct. For each target concept, a part feature was chosen that was high in distinctiveness (values of 0.5 and 1) and low in distinctiveness (values < 0.5). The unrelated conditions were created by re-pairing the distinctive and non-distinctive distractor words with unrelated targets following Costa et al. (2005; Experiment 2). Thus, each picture appeared four times, and each distractor word was used twice (with the exception of *stem* that was paired four times with different pictures due to a clerical error; as the results reported below did not differ when this item was removed from analyses, it was retained). Distinctive and non-distinctive distractors were also matched on a number of lexical variables including length, frequency, number of syllables and phonemes, OLD and PLD and word mean bigram frequency (Balota et al., 2007), age of acquisition (Kuperman et al., 2012), and concreteness (Brysbaert et al., 2013), summarized in Table 3. None of the objects were associates (probabilities < 0.01 in either direction) according to the University of South Florida Free Association Norms (Nelson et al., 2004) and Edinburgh Associative Thesaurus (Kiss et al., 1973). Following Costa et al. (2005; p. 127), the part of the object to which the distractor referred was not visible in the target picture (see Figure 2 for examples). There were no significant differences between distinctive and non-distinctive distractors on word length *t*_(46)_ = 0.12, *p* = 0.91, frequency *t*_(46)_ = 0.09, *p* = 0.93, OLD *t*_(46)_ = 0.64, *p* = 0.52, PLD *t*_(46)_ = 0.79, *p* = 0.44, number of phonemes *t*_(46)_ = 0.50, *p* = 0.62, number of syllables *t*_(46)_ = 0.32, *p* = 0.75, number of morphemes *t*_(46)_ = 0.59, *p* = 0.56, bigram frequency *t*_(46)_ = 1.04 *p* = 0.31, age of acquisition *t*_(46)_ = 0.30, *p* = 0.77, imageability *t*_(46)_ = 1.21 *p* = 0.23 and concreteness *t*_(46)_ = 0.45, *p* = 0.65.

**Table 3:** **Matching variables for the stimuli in Experiment 2**.

	**Distractor type**	
	**Distinctive**	**Non-distinctive**
Distinctiveness	0.88 (0.23)	0.14 (0.09)
Length	4.92 (1.32)	4.88 (1.12)
Frequency	34.09 (62.59)	35.54 (54.06)
OLD	1.59 (0.52)	1.68 (0.44)
PLD	1.50 (0.66)	1.38 (0.42)
Bigram frequency	1715.03 (914.47)	1982.26 (873.99)
Phonemes	3.96 (1.20)	3.79 (1.14)
Syllables	1.25 (0.44)	1.29 (0.46)
Age of acquisition	6.40 (2.02)	6.30 (2.35)
Imageability	5.70 (0.69)	6.01 (0.98)
Concreteness	4.64 (0.34)	4.69 (0.48)
Morphemes	1.08 (0.28)	1.04 (0.20)

*Standard deviations are in parentheses*.

*OLD, Orthographic Levenshtein Distance; PLD, Phonological Levenshtein Distance*.

### Procedure

The pre-experimental familiarization and experimental trial delivery were identical to Experiment 1. Participants completed three blocks of picture naming trials, one block at each SOA, with a brief rest period between each block. Participants viewed each picture paired with three distractor types (distinctive, non-distinctive, and unrelated) at each SOA. The order of the trials within each block was pseudorandomized across participants using Mix software (van Casteren and Davis, 2006) such that two presentations of the same picture were always interceded by at least five different pictures, and no more than two consecutive trials were of the same distractor type. The order of the three SOA blocks was counterbalanced across participants according to a Latin square design.

### Results and discussion

Data from two participants were excluded as they failed to trigger the voice key on > 50% of trials, leaving a final *N* = 27. Trials on which the voice key failed to detect a response (<1%) were discarded as were latencies below 250 ms or above 2000 ms (0.5%). Latencies deviating more than 2.5 standard deviations from within-participant, within-condition means were excluded from analysis (3.1%). Errors were classified according to whether the participant hesitated during naming (i.e., dysfluencies) or misidentified the target, and due to their low frequency (0.4%) were not subjected to analysis.

Data was subjected to a repeated-measures ANOVA by participants and by items, denoted as *F*_1_ and *F*_2_, respectively. Mean naming latencies, 95% *CI*s and error rates are summarized in Table 4. There were no significant effects of distractor part-relation, *F*_1(1, 26)_ < 1, *p* = 0.705, partial η^2^ = 0.01, *F*_2(1, 23)_ < 1, *p* = 0.659, partial η^2^ = 0.01, or distinctiveness, *F*_1(1, 26)_ < 1, *p* = 0.462, partial η^2^ = 0.02, *F*_2(1, 23)_ < 1, *p* = 0.438, partial η^2^ = 03. There was also no significant effect of SOA by participants *F*_1(2, 52)_ = 1.88, *p* = 0.163, partial η^2^ = 0.07, although the effect was significant by items, *F*_2(2, 46)_ = 4.56, *p* = 0.016, partial η^2^ = 0.17. As Table 4 shows, naming latencies were faster overall at the −150 ms SOA. There was no significant interaction between distractor part-relation and distinctiveness, *F*_1(2, 52)_ < 1, *p* = 0.774, partial η^2^ = 0.00, *F*_2(2, 46)_ < 1, *p* = 0.743, partial η^2^ = 0.01. In addition, there was no significant part-relation × SOA interaction, *F*_1(2, 52)_ < 1, *p* = 0.905, partial η^2^ = 0.00, *F*_2(2, 46)_ < 1, *p* = 0.772, partial η^2^ = 0.01. There was also no significant distinctiveness × SOA interaction, *F*_1(2, 52)_ < 1, *p* = 0.716, partial η^2^ = 0.01, *F*_2(2, 46)_ < 1, *p* = 0.894, partial η^2^ = 0.01. Finally, there was no significant three-way interaction between distractor relation, distinctiveness, and SOA, *F*_1(2, 52)_ < 1, *p* = 0.698, partial η^2^ = 0.01, *F*_2(2, 46)_ < 1, *p* = 0.918, partial η^2^ = 0.00.

**Table 4:** **Experiment 2: Naming Latencies (in Milliseconds), 95% Confidence Intervals (CI), and Error rates (E%) by Type of Distractor and SOA**.

	**Distractor condition**			
	**Distinctive**	**Non-distinctive**	**Unrelated (distinctive)**	**Unrelated (non-distinctive)**
**SOA −150**				
Mean	655	656	654	652
*CI*	±20	±20	±21	±20
*E%*	0.2	0.6	0.3	0.3
**SOA 0**				
Mean	665	669	665	665
*CI*	±21	±22	±23	±24
*E%*	0.3	0.7	0.5	0.3
**SOA +150**				
Mean	661	662	659	659
*CI*	±25	±23	±24	±19
*E%*	0.2	0.5	0.3	0.3

The results of Experiment 2 can be summarized as follows: part-whole distractor-target relations did not influence naming latencies compared to unrelated parts, irrespective of whether the part was a distinctive feature of the depicted object. The failure to observe an effect of part-whole relatedness is inconsistent with the results of Costa et al. (2005; also Muehlhaus et al., 2013), although consistent with the findings of Sailor and Brooks (2014; Experiments 2 and 3) for non-associate parts at the same SOAs. Thus, associative strength might be a confounding factor for reports of facilitation effects with part-whole relations as proposed by Piai et al. (2011; see also Sailor and Brooks, 2014).

However, it is possible that our failure to obtain an effect of feature distinctiveness for related part distractors reflects the manner in which the stimuli were constructed. Following Costa et al. (2005), the part of the object to which the distractor referred was not visible in the target picture (cf., Sailor and Brooks, 2014, Experiment 2). Feature-distinctiveness effects have been reported in basic level picture naming (e.g., Taylor et al., 2012). As Cree et al. (2006) note, in such tasks it is beneficial to *recognize* a visual feature that is unique to the target. Accordingly, we conducted Experiment 3 to test whether feature distinctiveness will influence picture naming latencies when the distractor refers to a part that is visible in the target object.

## Experiment 3

Experiment 3 tests whether feature distinctiveness will influence picture naming latencies in the PWI paradigm when the distractor refers to a part that is visible in the target object.

### Participants

Participants were 27 students from the University of Queensland. All were native English speakers. Each participant gave informed consent and was compensated with course credit.

### Research design

The design was identical to Experiment 2.

### Materials

Materials were constructed in an identical manner to Experiment 2, although the features that the related-part distractors referred to were now visible in the respective target pictures (see Appendix C). In order to ensure feature visibility, some of the non-distinctive items used in Experiment 2 were replaced. Distinctive and non-distinctive distractors were also matched on a number of lexical variables (see Table 5) including length, frequency, number of syllables and phonemes, OLD and PLD and word mean bigram frequency (Balota et al., 2007), age of acquisition (Kuperman et al., 2012), and concreteness (Brysbaert et al., 2013). None of the objects were associates (probabilities < 0.01 in either direction) according to the University of South Florida Free Association Norms (Nelson et al., 2004) and Edinburgh Associative Thesaurus (Kiss et al., 1973). There were no significant differences between distinctive and non-distinctive part distractors on word length *t*_(46)_ = 1.57, *p* = 0.12, frequency *t*_(46)_ = 0.10, *p* = 0.92, OLD *t*_(46)_ = 0.31, *p* = 0.76, PLD *t*_(46)_ = 1.63, *p* = 0.11, number of phonemes *t*_(46)_ = 0.1.41, *p* = 0.16, number of syllables *t*_(46)_ = 1.42, *p* = 0.16, bigram frequency *t*_(46)_ = 0.49, *p* = 0.63, age of acquisition *t*_(46)_ = 1.90, *p* = 0.06, imageability *t*_(46)_ = 1.14, *p* = 0.26 and concreteness *t*_(46)_ = 1.08, *p* = 0.28.

**Table 5:** **Matching variables for the stimuli in Experiment 3**.

	**Distractor type**	
	**Distinctive**	**Non-distinctive**
Distinctiveness	0.88 (0.23)	0.12 (0.09)
Length	5.00 (1.32)	4.50 (1.06)
Frequency	30.89 (60.74)	30.69 (41.17)
OLD	1.63 (0.51)	1.62 (0.40)
PLD	1.53 (0.65)	1.31 (0.32)
Bigram frequency	1706.76 (914.38)	1822.09 (948.50)
Phonemes	4.00 (1.18)	3.58 (1.06)
Syllables	1.29 (0.46)	1.13 (0.34)
Age of acquisition	6.48 (1.91)	5.47 (1.84)
Imageability	5.69 (0.68)	5.96 (0.92)
Concreteness	4.61 (0.34)	4.73 (0.46)

*Standard deviations are in parentheses*.

*OLD, Orthographic Levenshtein Distance; PLD, Phonological Levenshtein Distance*.

### Procedure

The procedure was identical to Experiment 2.

### Results and discussion

Trials on which the voice key failed to detect a response (<1%) were discarded as were latencies below 250 ms or above 2000 ms (<1%). Latencies deviating more than 2.5 standard deviations from within-participant, within-condition means were excluded from analysis (3.2%). Errors were classified according to whether the participant hesitated during naming (i.e., dysfluencies) or misidentified the target, and due to their low frequency (1.2%) were not subjected to analysis. Data were subjected to repeated-measures ANOVAs by participants and by items. Means, CIs, and error rates are reported in Table 6.

**Table 6:** **Experiment 3: Naming Latencies (in Milliseconds), 95% Confidence Intervals (CI), and Error rates (E%) by Type of Distractor and SOA**.

	**Distractor condition**			
	**Distinctive**	**Non-distinctive**	**Unrelated (distinctive)**	**Unrelated (non-distinctive)**
**SOA −150**				
Mean	634	636	633	622
*CI*	±26	±25	±24	±28
*E%*	2	2	0.6	1.4
**SOA 0**				
Mean	634	638	639	635
*CI*	±29	±29	±33	±32
*E%*	1.5	1.7	1.4	1.1
**SOA +150**				
Mean	641	634	636	640
*CI*	±32	±31	±29	±29
*E%*	1.1	1.4	0.5	0.9

The main effect of distractor part-relation was not significant, *F*_1(1, 26)_ < 1, *p* = 0.428, partial η^2^ = 0.02, *F*_2(1, 23)_ < 1, *p* = 0.480, partial η^2^ = 0.02. There was also no significant main effect of distinctiveness, *F*_1(1, 26)_ < 1, *p* = 0.333, partial η^2^ = 0.04, *F*_2(1, 23)_ < 1, *p* = 0.330, partial η^2^ = 0.04. Although the main effect of SOA was not significant by participants, *F*_1(2, 52)_ < 1, *p* = 0.438, partial η^2^ = 0.03, it was marginally significant by items *F*_2(2, 46)_ = 3.15, *p* = 0.052, partial η^2^ = 0.12. The interaction between distractor part-relation and distinctiveness was not significant, *F*_1(1, 26)_ < 1, *p* = 0.515, partial η^2^ = 0.02, *F*_2(1, 23)_ = 1.31, *p* = 0.264, partial η^2^ = 0.05. This was also the case for the part-relation × SOA interaction, *F*_1(2, 52)_ = 2.02, *p* = 0.144, partial η^2^ = 0.07, *F*_2(2, 46)_ = 1.72, *p* = 0.190, partial η^2^ = 0.07, and distinctiveness × SOA interaction, *F*_1(2, 52)_ < 1, *p* = 0.576, partial η^2^ = 0.02, *F*_2(2, 46)_ < 1, *p* = 0.649, partial η^2^ = 0.02. However, the three-way interaction between distractor part-relation, distinctiveness and SOA was marginally significant, *F*_1(2, 52)_ = 2.97, *p* = 0.060 partial η^2^ = 0.10, *F*_2(2, 46)_ = 2.70, *p* = 0.078, partial η^2^ = 0.11.

Additional analyses investigated the three-way interaction. At −150 ms SOA, there was a significant effect of part-relation by participants, *F*_1(1, 26)_ = 8.46, *p* = 0.007 partial η^2^ = 0.25, but was only marginally significant by items *F*_2(1, 23)_ = 3.77, *p* = 0.065, partial η^2^ = 0.14. There was no significant effect of distinctiveness *F*_1(1, 26)_ = 1.41, *p* = 0.246, partial η^2^ = 0.05, *F*_2(1, 23)_ = 1.57, *p* = 0.225, partial η^2^ = 0.06 or interaction by participants *F*_1(1, 26)_ = 2.64, *p* = 0.116, partial η^2^ = 0.09, however the interaction was significant by items *F*_2(1, 23)_ = 5.96, *p* = 0.023, partial η^2^ = 0.21. At 0 ms SOA, there was no significant effect of relatedness *F*_1(1, 26)_ < 1, *p* = 809, partial η^2^ = 0.00, *F*_2(1, 23)_ < 1, *p* = 0.716, partial η^2^ = 0.01, no significant effect of distinctiveness *F*_1(1, 26)_ < 1, *p* = 0.946, partial η^2^ = 0.00, *F*_2(1, 23)_ < 1, *p* = 0.884, partial η^2^ = 0.00, and no interaction *F*_1(1, 26)_ < 1, *p* = 0.342, partial η^2^ = 0.04, *F*_2(1, 23)_ < 1, *p* = 0.397, partial η^2^ = 0.03. At 150 ms SOA, there was no significant effect of relatedness *F*_1(1, 26)_ < 1, *p* = 0.775, partial η^2^ = 0.00, *F*_2(1, 23)_ < 1, *p* = 0.762, partial η^2^ = 0.00, no significant effect of distinctiveness *F*_1(1, 26)_ < 1, *p* = 0.517, partial η^2^ = 0.02, *F*_2(1, 23)_ < 1, *p* = 0.664, partial η^2^ = 0.01, and no interaction *F*_1(1, 26)_ = 2.38, *p* = 0.135, partial η^2^ = 0.08, *F*_2(1, 23)_ = 2.54, *p* = 0.125, partial η^2^ = 0.10.

Paired-samples *t*-tests were conducted to investigate the significant effects found at −150 ms SOA. There were no significant differences between distinctive and non-distinctive distractors *t*_1(26)_ = 0.329, *p* = 0.744, *M*_diff_ = 2 ms, 95% *CI* = ±11, *t*_2(23)_ = 0.543, *p* = 0.592, *M*_diff_ = 3 ms, 95% *CI* = ±10 or between distinctive and unrelated distractors *t*_1(26)_ = 0.355, *p* = 0.741, *M*_diff_ = 1 ms, 95% *CI* = ±9, *t*_2(23)_ = 0.228, *p* = 0.822, *M*_diff_ = 1 ms, 95% *CI* = ±12. However, there was a significant difference between non-distinctive distractors *t*_1(26)_ = 2.727, *p* = 0.011, *M*_diff_ = 14 ms, 95% *CI* = ±11, *t*_2(23)_ = 3.383, *p* = 0.003, *M*_diff_ = 16 ms, 95% *CI* = ±10 such that non-distinctive distractors were named more slowly than unrelated distractors.

The results of Experiment 3 differ from Experiment 2, in that *non-distinctive* part-whole target-distractor relations slowed picture naming latencies significantly at −150 ms SOA compared to their matched unrelated pairings. This is consistent with the results of Sailor and Brooks (2014, Experiments 1 and 3) who reported interference from non-associated parts.

## General discussion

In three experiments using the PWI paradigm, we investigated the roles of distinctive vs. shared conceptual features in lexical access. Experiment 1 employed categorically-related distractor-target pairings manipulated in terms of the presence/absence of a distinctive feature. Experiments 2 and 3 manipulated part-whole related distractor-target pairings in terms of distinctive vs. non-distinctive features and in terms of feature visibility in the target pictures. In Experiments 1 and 2, feature distinctiveness did not influence picture naming latencies differentially. In Experiment 3, non-distinctive part distractors that were visible in the target pictures slowed picture naming latencies significantly compared to their matched unrelated distractors at SOA −150 ms.

Experiment 1 indicates that the presence of a distinctive feature in categorically-related distractor-target pairings does not influence picture naming when those pairings are matched in terms of conceptual feature overlap. Semantically similar-plus-distinctive distractors slowed picture naming to the same degree as semantically similar distractors *without* a distinctive feature. Therefore, it seems unlikely that distinctive features can explain some facilitation results reported with categorically-related, semantically-close stimuli (Mahon et al., 2007). As we tested more participants (25 at each SOA) than Mahon et al. (2007; 20 and 16 at each SOA in their Experiments 5 and 7, respectively), the null effects are unlikely to be due to lack of power. Why is it that distinctive features facilitate basic-level naming (Grondin et al., 2009; Taylor et al., 2012) and produce priming relative to shared features in word-feature verification tasks (e.g., Cree et al., 2006), yet do not influence naming latencies in PWI? Grondin et al. were careful to emphasize the importance of task variables for determining the relative contributions of distinctive vs. shared features to performance. In Experiment 1, both types of distractor also shared many features with the target. This suggests that distinctive feature activation does not predominate in the presence of activation from many shared features (e.g., Cree et al., 2006), and so does not influence production of the target name. This finding can be accommodated by existing competitive lexical selection (Vigliocco et al., 2004; Rahman and Melinger, 2009; Vieth et al., 2014) and response exclusion accounts (e.g., Mahon et al., 2007). In the former, feature overlap predominates and activates a lexical cohort with the net result being competition; in the latter, identical response relevant criteria result in the post-lexical decision mechanism taking more time to clear both types of distractor from the articulatory buffer.

Experiments 2 and 3 manipulated distinctive features to investigate the part-whole facilitation effect reported by Costa et al. (2005). In Experiment 2, the part distractors were not visible in the target picture in keeping with Costa et al.'s (2005; p. 127) materials. Following proposals that distinctive features need to be visible in order to influence picture naming (Grondin et al., 2009), Experiment 3 ensured that the part the distractor referred to was visible in the target picture. In Experiment 2, we failed to find *any* effect of part-whole related compared to their matched unrelated part distractors. However, when the part denoted by a distractor was visible in the target (Experiment 3), only *non-distinctive* parts slowed picture naming latencies significantly compared to their matched unrelated parts. Sailor and Brooks (2014; Experiment 2) were unable to replicate the facilitation effect reported by Costa et al.'s (2005) Experiment 2 with the same materials and procedure (but see Discussion re part visibility below). However, they demonstrated significant interference with non-associated part distractors in two other experiments.

The results of Experiment 3 are therefore broadly consistent with those of Sailor and Brooks' (2014), in that we also observed interference with non-associated parts. However, they also add to this finding by demonstrating that non-associated part distractors are likely to slow naming latencies in PWI *only* if they do not denote a distinctive feature of the target picture concept. These findings can be accommodated by the lexical selection by competition account. According to this account, activation should spread from the target (e.g., GOAT) to the part distractor (e.g., *tail*). As non-distinctive parts are shared by many category exemplars (e.g., most animals have *tails*), spreading activation should therefore result in greater competition at the lexical level. By contrast, as the target spreads activation only to the distinctive part (e.g., *beard*), less lexical competition occurs due to the one-to-one mapping (see Figure 1). A caveat to this interpretation is that the mean naming latencies for distinctive vs. non-distinctive part distractors did not differ significantly^1^. Interestingly, this was the same pattern reported for the mean naming latencies in Experiments 1 and 3 of Sailor and Brooks (2014), i.e., naming latencies for their associated and non-associated part-related distractors were comparable (see their Tables 1, 3). Nonetheless, the principal comparisons of interest are between each type of related part and their identically matched unrelated distractors. Although the distinctive and non-distinctive distractor words were matched on a range of variables (see Table 5), they were not matched identically as was the case with their respective unrelated distractors.

The results of Experiments 2 and 3 also highlight a potentially important role for feature visibility in determining whether interference will be observed. In conventional PWI experiments with categorically-related distractors, object features are typically visible in the target picture. According to the lexical selection by competition account, the target concept spreads activation to the related distractor due to feature overlap, raising its activation level and that of other lexical competitors. This might explain why distractors denoting visible non-distinctive parts interfered with target picture naming (Experiment 3), compared to non-visible parts (Experiment 2). Cree et al. (2006) had earlier proposed that a feature must be *recognized* in the target object in order for it to be beneficial to picture naming. In terms of PWI, this suggests the target picture concept is able to spread activation to the part distractor once the part is recognized, and this activation then spreads to the lexical level. Thus, feature visibility might be an important factor determining whether interference effects will be elicited with part distractors, and whether facilitation will predominate when associative relations are also present. For example, Costa et al. (2005; Experiment 2) ensured the parts denoted by their distractors (many of which were distinctive *and* associates) were not visible in the target pictures, whereas Sailor and Brooks' (2014) replication of Costa et al.'s experiment did not.

The findings of interference for part-whole related distractors have implications for recently formulated models of lexical access and PWI effects (see Sailor and Brooks, 2014). Both the response exclusion (Mahon et al., 2007) and swinging lexical network (Rahman and Melinger, 2009) accounts were developed to explain reports of semantic facilitation that were deemed problematic for the conventional lexical selection by competition account. Following those earlier reports, both accounts assumed that part distractors facilitate whole object naming via semantic priming. However, it seems that facilitation effects for part distractors in PWI might not be reproducible, unless parts also have an associative relation with the target picture, as proposed by Piai et al. (2011; e.g., Muehlhaus et al., 2013; Sailor and Brooks, 2014). Facilitation with associative part relations can be accounted for by a competitive lexical selection model by assuming the effect occurs at the conceptual level (see La Heij et al., 1990, 2006). One possible way of modifying the response exclusion account to explain the interference effect observed in Experiment 3 might involve making the additional assumption that visible features of target pictures constitute response relevant criteria, despite the instruction to name the whole object (see also Sailor and Brooks, 2014). However, adopting this modification would first involve abandoning Mahon et al.'s (2007) proposal that conceptual feature overlap does not constitute a response-relevant criterion.

Theoretical accounts of PWI effects have emphasized the semantic relationship between concepts as the determining factor of an effect. However, experimental evidence shows that wide ranges of effects are possible for each type of relationship (i.e., categorical, associative, part-whole). This suggests that variables other than semantic relationship can influence the polarity of PWI effects, and that other reports of semantic facilitation in PWI might be due to task and/or procedural factors. For example, in their Experiment 1, Costa et al. (2005) compared part distractors (e.g., LAMP-*bulb*) to categorical, but unrelated distractors (e.g., LAMP-*wolf*) rather than part distractors at the same level of categorization as in the present and other studies (e.g., Sailor and Brooks, 2014). Costa et al. (2003) had earlier argued that the level of categorization could be used by the semantic system to differentiate the conceptual representations corresponding to the target and distractor. According to their semantic selection account, when target and distractor are from different levels of categorization the semantic system discards the distractor's conceptual representation for further processing, preventing lexical competition from arising. However, the distractor's conceptual representation will enhance the activation of the target, leading to semantic facilitation (but see Kuipers et al., 2006; Hantsch et al., 2012).

Although semantic facilitation in PWI has proved difficult to reproduce in the absence of associative relations, a study by Collina et al. (2013) suggests picture familiarization might also be a possible cause of semantic polarity reversals in PWI. In most PWI studies, participants are typically familiarized with the target pictures two-to-four times prior to performing the experimental series, as was the case in the present study (e.g., Starreveld and La Heij, 1995, 1996; Damian and Martin, 1999; Mahon et al., 2007). In Collina et al.'s study, participants who were familiarized with the target pictures showed interference compared to those who were not familiarized with the target pictures while the latter group showed facilitation. Given that a picture familiarization phase is a standard procedure in PWI experiments (e.g., Starreveld and La Heij, 1995, 1996; Mahon et al., 2007), Collina et al.'s (2013) finding warrants replication and further investigation.

In summary, our findings do not provide empirical support for the proposal that part-whole distractor-target relations facilitate naming in PWI via semantic priming (cf. Costa et al., 2005; Mahon et al., 2007), unless an associative relation is also involved (e.g., Piai et al., 2011; Muehlhaus et al., 2013; Sailor and Brooks, 2014). Instead, our findings indicate that an interference effect can be observed when a non-associated part distractor denotes a conceptual feature shared by the target and other category exemplars. This activation appears contingent on the feature denoted by the part distractor being visible in the target picture. Distinctive features did not influence the level of lexical activation significantly. Together, these findings indicate that semantic interference effects in the PWI paradigm are a product of conceptual feature overlap, consistent with the assumptions of prominent lexical selection by competition accounts (e.g., Roelofs, 1992; Starreveld and La Heij, 1995, 1996; Levelt et al., 1999).

### Conflict of interest statement

The authors declare that the research was conducted in the absence of any commercial or financial relationships that could be construed as a potential conflict of interest.

## Footnotes

^1^We are grateful to an anonymous reviewer for drawing our attention to this.
