# Supplementary material for: The roles of shared vs. distinctive conceptual features in lexical access
Source: Front Psychol. 2014 Sep 16;5:1014. doi: 10.3389/fpsyg.2014.01014 (PMC4165322; doi:10.3389/fpsyg.2014.01014)
Supplement: Supplementary file 1 [file DataSheet1.DOCX]

Appendix A.

Materials for Experiment 1. Distinguishing feature dimension in parentheses.

|  | Distractor Condition | | | |
| --- | --- | --- | --- | --- |
| Target Picture | Similar | Similar-plus-distinguishing | Unrelated | Unrelated |
|  |  |  |  |  |
| BACKPACK | satchel | suitcase (handle) | adder | cobra |
| BENCH | chair | stool (back) | sparrow | canary |
| BOLT | screw | nail (thread) | swordfish | tuna |
| CABINET | pantry | wardrobe (room) | porpoise | whale |
| CAN | barrel | bucket (handle) | jumper | vest |
| CAPSULE | pill | tablet (gel casing) | stork | pelican |
| COAT | jumper | vest (sleeves) | barrel | bucket |
| DOLPHIN | porpoise | whale (size) | chair | stool |
| HOE | shovel | rake (tines) | pantry | wardrobe |
| IBIS | stork | pelican (throat pouch) | pill | tablet |
| LEOPARD | cheetah | panther (spots) | shovel | rake |
| MARLIN | swordfish | tuna (long bill) | screw | nail |
| PIGEON | sparrow | canary (yellow plumage) | bugle | horn |
| PYTHON | adder | cobra (hood) | bus | truck |
| STAMP | label | ticket (adhesive) | cart | carriage |
| TRUMPET | bugle | horn (valves) | cheetah | panther |
| TURKEY | pheasant | peacock (tail) | satchel | suitcase |
| VAN | bus | truck (cargo area) | pheasant | peacock |
| WAGON | cart | carriage (body) | cucumber | Eggplant |
| ZUCCHINI | cucumber | eggplant (colour) | label | ticket |
|  |  |  |  |  |

Note: Data from the two unrelated conditions were averaged.

Appendix B

Materials for Experiment 2

|  | Distractors | | | |
| --- | --- | --- | --- | --- |
| Target Picture | Distinctive | Non-distinctive | Unrelated | Unrelated |
| BAT | Fangs | Stomach | Pin | Cord |
| BED | Springs | Foam | Fangs | Eye |
| BRA | Hook | Cloth | Stone | Ceiling |
| CAMEL | Hump | Knee | Hole | Floor |
| CHURCH | Altar | Seat | Liver | Talons |
| CLOCK | Face | Spindle | Knee | Udder |
| COTTAGE | Fireplace | Floor | Snout | Stomach |
| COW | Udder | Liver | Rack | Plug |
| CROCODILE | Jaws | Heart | Fret | Bowl |
| DISHWASHER | Rack | Hose | Face | Tongue |
| DUCK | Bill | Eye | Cable | Spindle |
| ELEPHANT | Trunk | Teeth | Foam | Springs |
| GOAT | Beard | Tail | Cloth | Fireplace |
| GRENADE | Pin | Lever | Beard | Tail |
| GUITAR | Hole | Fret | Hump | Bone |
| LAMP | Switch | Cord | Teeth | Jaws |
| ELEVATOR | Cable | Ceiling | Stem | Core |
| MISSILE | Warhead | Engine | Altar | Seat |
| MIXER | Bowl | Plug | Heart | Trunk |
| MOUSE | Ball | Sensor | Bill | Stem |
| PEACH | Stone | Stem | Engine | Warhead |
| PIG | Snout | Tongue | Sensor | Switch |
| PINEAPPLE | Core | Stone | Lever | Hook |
| VULTURE | Talons | Bone | Hose | Ball |

Appendix C

Materials for Experiment 3

|  | Distractors | | | |
| --- | --- | --- | --- | --- |
| Target Picture | Distinctive | Non-distinctive | Unrelated | Unrelated |
| BAT | Fangs | Stomach | Pin | Cord |
| BED | Springs | Foam | Fangs | Eye |
| BRA | Hook | Cloth | Stone | Ceiling |
| CAMEL | Hump | Knee | Floor | Hole |
| CHURCH | Altar | Seat | Skin | Talons |
| CLOCK | Face | Glass | Udder | Knee |
| COTTAGE | Fireplace | Floor | Stomach | Snout |
| COW | Udder | Skin | Rack | Plug |
| CROCODILE | Jaws | Scales | Fret | Bowl |
| DISHWASHER | Rack | Latch | Face | Wings |
| DUCK | Bill | Eye | Glass | Cable |
| ELEPHANT | Trunk | Toe | Foam | Springs |
| ELEVATOR | Cable | Ceiling | Core | Stem |
| GOAT | Beard | Tail | Cloth | Fireplace |
| GRENADE | Pin | Lever | Tail | Beard |
| GUITAR | Hole | Fret | Hair | Hump |
| LAMP | Switch | Cord | Jaws | Toe |
| MISSILE | Warhead | Fin | Seat | Altar |
| MIXER | Bowl | Plug | Scales | Trunk |
| MOUSE | Button | Wheel | Bill | Leaf |
| PEACH | Stone | Stem | Warhead | Fin |
| PIG | Snout | Hair | Wheel | Switch |
| PINEAPPLE | Core | Leaf | Hook | Lever |
| VULTURE | Talons | Wings | Latch | Button |
